# The Impact of Water-Soluble Chitosan on the Inhibition of Crystal Nucleation of Alpha-Mangostin from Supersaturated Solutions

**DOI:** 10.3390/polym14204370

**Published:** 2022-10-17

**Authors:** Arif Budiman, Nisrina Nurfadilah, Muchtaridi Muchtaridi, Sriwidodo Sriwidodo, Diah Lia Aulifa, Agus Rusdin

**Affiliations:** 1 Department of Pharmaceutics and Pharmaceutical Technology, Faculty of Pharmacy, Universitas Padjadjaran, Jl. Raya Bandung-Sumedang Km. 21, Bandung 45363, Indonesia; 2 Department of Pharmaceutical Analysis and Medicinal Chemistry, Faculty of Pharmacy, Universitas Padjadjaran, Jl. Raya Bandung-Sumedang Km. 21, Bandung 45363, Indonesia

**Keywords:** supersaturation, polymers, amorphous, crystallization, alpha-mangostin, water-soluble chitosan

## Abstract

The use of an amorphous drugs system to generate supersaturated solutions is generally developed to improve the solubility and dissolution of poorly soluble drugs. This is because the drug in the supersaturation system has a high energy state with a tendency to precipitate. In the amorphous solid dispersion (ASD) formulation, it was discovered that polymer plays a critical role in inhibiting nucleation or crystal growth of the drugs. Therefore, this study aimed to evaluate the crystallization inhibition of water-soluble chitosan (WSC) on nucleation as well as crystal growth from alpha-mangostin (AM) and elucidate its inhibition mechanism in the supersaturated solutions. During the experiment, WSC was used as a polymer to evaluate its ability to inhibit AM nucleation. The interaction between WSC and AM was also estimated using FT-IR, NMR, and in silico study. The result showed that in the absence of polymer, the concentration of AM rapidly decreased due to the precipitation in one minute. Meanwhile, the addition of WSC effectively inhibited AM crystallization and maintained a supersaturated state for the long term. FT-IR measurement also revealed that the shift in the amine primer of WSC occurred because of the interaction between WSC and AM. In the ^1^H NMR spectra, the proton peaks of WSC showed an upfield shift with the presence of AM, indicating the intermolecular interactions between AM and WSC. Moreover, in silico study revealed the hydrogen bond interaction between the carbonyl group of AM with hydrocarbon groups of WSC. This indicated that WSC interacted with AM in the supersaturated solution and suppressed their molecular mobility, thereby inhibiting the formation of the crystal nucleus. Based on these results, it can be concluded that the interaction between drug polymers contributed to the maintenance of the drug supersaturation by inhibiting both nucleation and growth.

## 1. Introduction

Recent investigations showed that over 70% of new drug candidates were poorly water-soluble. This is because their bioavailability is very low when developed as oral drug formulations and sometimes abandoned due to insufficient absorption [1,2]. Therefore, the development of a strategy to improve the solubility is necessary to formulate poorly water-soluble drugs, specifically for oral drug formulation [3,4]. In recent years, various strategies have been developed to improve solubility such as amorphous solid dispersions [4,5], cocrystals, and [6,7] co-amorphous systems [8,9]. These formulation techniques can create the drug in the supersaturation state [10].

Supersaturation of drugs has been extensively investigated as an important study area in drug delivery. This is due to an increase in the solubility of poorly water-soluble drugs, which directly contributes to improving absorption [11]. When these drugs dissolve in the water, their concentrations will be at supersaturated levels. This indicated that the concentration of the dissolved drug is higher than that of the equilibrium solubility of crystalline free forms [12]. However, the drug in the supersaturation state is thermodynamically unstable, and the concentration of the dissolved drug quickly decreases due to recrystallization [13]. This makes an additional excipient very important to stabilize the supersaturated state of the drug and inhibit the crystallization of stable forms [10].

Nucleation and crystal growth are the two steps in the crystallization of supersaturated drugs. To maintain the supersaturation or prevent precipitation/crystallization, one or both steps need to be inhibited. Several kinds of excipients that have been investigated to inhibit the crystallization of drug in supersaturated solutions include cyclodextrins, surfactants, and water-soluble polymers, which are often used as crystallization inhibitors [14,15]. These excipients can prevent precipitation in solution, delay crystallization by inhibiting nucleation or crystal growth, and maintain supersaturation of the drug for a long time. Polymers such as methacrylate copolymers [16,17], hypromellose (HPMC) [18,19], and polyvinylpyrrolidone (PVP) [20,21] that were used as carriers in the amorphous solid effectively inhibited the recrystallization of the drug in supersaturated solutions. This was carried out to achieve higher and longer drug supersaturation on oral administration. Recrystallization of drug molecules, drug–excipient/polymer interactions, and molecular mobility also play a significant role in the inhibition of drug precipitation from the supersaturated system [12].

Chitosan is a polymer that can easily form amorphous solid dispersion using a casting and a spray-drying technique. It is a cationic natural biopolymer that is produced by the alkaline *N*-deacetylation of chitin. Furthermore, it has been an interesting material in pharmaceutical formulation due to its biocompatibility [22], biodegradability [23], and low toxicity. Chitosan is insoluble in water or organic solvent; thus, various methods have been conducted to enhance its solubility. A previous study reported that water-soluble chitosan (WSC) can be produced by deacetylation of chitin [24]. Thus, WSC can form supersaturated solutions in amorphous drug formulation after dissolving in the water [25].

This study aims to elucidate the mechanism of WSC inhibition on the crystal nucleation of alpha-mangostin (AM) from supersaturated solutions. The AM was used as a model of the poorly water-soluble drug because it is an active compound sourced from herbal plants that can be used as an alternative drug, specifically for breast cancer treatment. AM is a xanthone derivative compound from the pericarp of the mangosteen fruit (*Garcinia mangostana* L.), which has pharmacological properties such as cardioprotective, anticancer, antioxidant, and anti-inflammatory [26]. However, it has poor aqueous solubility and low oral bioavailability [26]. Some reports have been carried out on the amorphous solid dispersion of AM, but the inhibition mechanism of WSC as a polymer on the crystal nucleation of AM from supersaturated solutions has not been investigated. There is no report on the use of WSC to inhibit the crystallization of AM and the relationship between AM–WSC interactions as well as their molecular mobility with precipitation inhibition of AM from the supersaturated system. Therefore, it is necessary to investigate the ability of WSC to inhibit the crystallization of AM from supersaturated solutions and develop the formulation of AM as poorly water-soluble drugs, especially for oral drugs.

## 2. Materials and Methods

### 2.1. Materials

**Figure 1:**
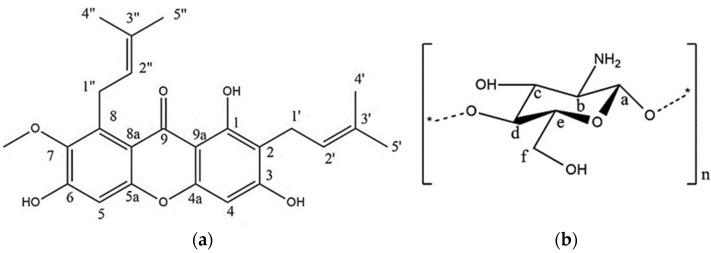
Chemical structures of (**a**) alpha-mangostin (AM) and (**b**) water-soluble chitosan (WSC).

AM (MW = 410.5 g/mol) was purchased from Chengdu Biopurify Phytochemicals (Shincuan, China), while water-soluble chitosan was purchased from Biochitosan Indonesia (Cirebon, Indonesia). The chemical structures of AM and WSC are shown in Figure 1. All chemicals were used as received without further purification.

### 2.2. Methods

#### 2.2.1. Powder X-ray Diffraction (PXRD) Measurement

The PXRD patterns were collected using a Kristalloflex Diffractometer D500 (Siemens, Berlin, Germany) with the following conditions, namely target Cu, filter Ni, voltage 40 kV, current 30 mA, scanning rate, 0.75°/min, and scanning angle of 2θ = 5–60°.

#### 2.2.2. Crystalline Solubility Measurements

The crystalline solubility of AM was determined using water containing 3% (*v*/*v*) DMSO, while WSC was dissolved in the water at various concentrations. Subsequently, an excess crystalline AM powder was added to the WSC solution and shaken for 48 h at 25 °C. The solutions were filtered through a 0.45 μm membrane filter, diluted with acetonitrile, and determined using high-performance liquid chromatography (HPLC).

#### 2.2.3. Nucleation Induction Time Measurements

WSC was dissolved in an aqueous solution at a concentration of 500 μg/mL. The AM supersaturated solutions were prepared by adding a stock solution of AM (1500 μg/mL in ACN) to the additive solutions, leading to a final ACN concentration of 2% (*v*/*v*) at 25 °C. The solutions were stirred at 150 rpm at 25 °C, filtered through a 0.45 μm membrane filter at different time points, diluted with acetonitrile, and determined using HPLC.

#### 2.2.4. HPLC Conditions

HPLC analysis was performed using a Dionex-Ultimate 3000 HPLC (Dionex, Sunnyvale, CA, USA) equipped with an Inertsil ODS C18 (6.0 × 150 mm) column at 30 °C. The mobile was composed of acetonitrile and 0.1% formic acid in water at a ratio of 95:5. The samples were analyzed by injecting 10 μL, the flow rate was set to 1 mL/min, and detected at 244 nm using a UV detector. Standard solutions of 1, 5, 10, 25, 50, and 100 μg/mL were prepared in the mobile phase, and the linear coefficient of determination (R2) value of AM standard solution was 0.9996.

#### 2.2.5. Fourier-Transform Infrared (FT-IR) Spectroscopy

Each sample conducted in nucleation induction time measurements was evaluated using a *Nicolet iS5 FT-IR spectrometer* (Thermo Scientific, Waltham, MA, USA) to determine the interaction between AM and WSC in an aqueous solution. The FT-IR spectra of each sample in an aqueous solution were obtained by subtracting the FT-IR spectrum of pure water as a blank value from each sample solution.

#### 2.2.6. NMR Measurements

WSC was dissolved in D2O and AM-supersaturated solutions were prepared by adding a stock solution of AM in DMSO-d6 to the additive solutions at 25 °C. Trimethylsilyl propionate (TSP) was used as an internal reference. The sample solution was measured using Bruker 500 MHZ NMR spectrophotometer (Billerica, MA, USA).

#### 2.2.7. In Silico Study

AM interactions with WSC were studied using a computer with the following specifications: Intel(R) Celeron(R) N4020 CPU @ 1.10 GHz 1.10 GHz, installed RAM 8.00 GB, 64-bit operating system, x64-based processor, Chemdraw, Discovery Studio v. 16.1.0 free trial (Dassault Systemes BIOVIA, San Diego, USA), and Autodock Tools (ADT) 1.5.6 software (The Scripps Research Institute, San Diego, CA, USA). AM and WSC were downloaded in two-dimensional (2D) type from www.Pubchem.ncbi.mlm.gov (accessed on 17 June 2022), or the structures were drawn using Chemdraw 2D, and the energy was reduced using MM2+ on Chemdraw 3D software. The structures were loaded into the Discovery Studio software version 16.1.0 (Dassault Systemes BIOVIA. San Diego, CA, USA) and saved as a PDB file. Subsequently, the interaction was observed using the ligand–ligand method to obtain the hydrogen bonding, binding energy (Ei), and distance of each interaction between AM and WSC.

#### 2.2.8. Viscosity Test

Viscosity measurements were carried out using Ametek Brookfield DVE viscometer with a volume sample of ±200 mL. The size of the spindle used was no. 61 with a rotation speed of 100 rpm.

## 3. Results

**Figure 2:**
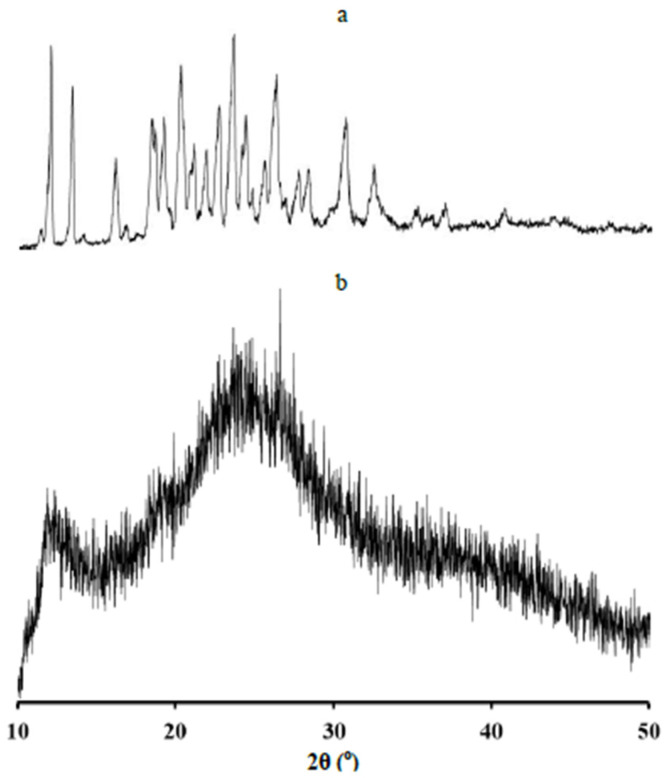
Characteristic diffraction peaks in PXRD of (**a**) alpha-mangostin (AM) and (**b**) water-soluble chitosan (WSC).

**Figure 3:**
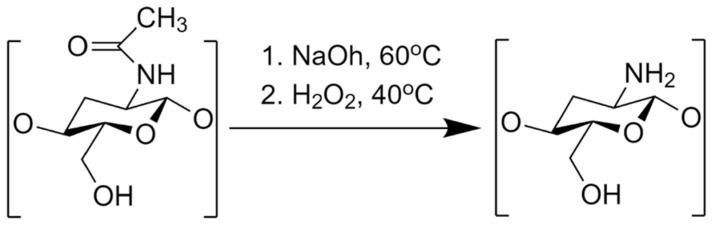
Synthesis of water-soluble chitosan (WSC).

The sample of AM showed the characteristic of diffraction peaks in the PXRD pattern, indicating its crystalline state, as presented in Figure 2. In contrast, WSC showed a halo pattern without any diffraction peaks indicating its amorphous state. WSC is insoluble in the water or the organic solvent and only soluble in an aqueous acidic solution. Chitosan is insoluble in the water or the organic solvent and only soluble in an aqueous acidic solution. The crystalline structure of chitosan is formed by strong hydrogen bonding between the hydroxyl group, acetamide group, and carbonyl group, inhibiting chitosan to be soluble in water [27]. However, WSC can be formed by demineralization and deproteination process, as shown in Figure 3. In our study, we used WSC as polymer because we investigated the crystallization inhibition of AM in the water. Thus, WSC used in the present study could be a potential inhibitor on the AM crystallization from supersaturated solutions.

### 3.1. Crystalline Solubility Measurements

**Figure 4:**
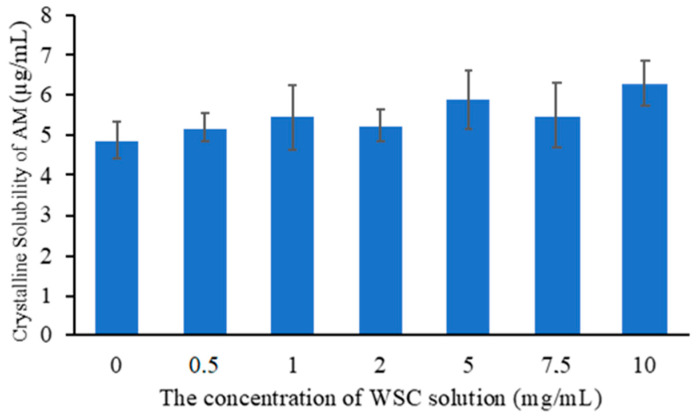
Crystalline solubility of AM at 25 °C in WSC solution with various concentrations.

Solubility measurement is very important in the selection of polymer concentration for the development of supersaturated drug systems because it can be an indicator of hydrophobicity [28]. The determination of the solubility of the drug in the presence of polymers is needed to confirm the impact of polymer on the thermal equilibrium solubility of the drug. The results of the solubility of AM crystal with the addition of WSC at various concentrations are presented in Figure 4, where the equilibrium solubility of the AM crystal was observed at 4.87 ± 0.45 μg/mL. When WSC with the concentrations of 0.5, 1, 2, 5, and 10 mg/mL was added, the thermal equilibrium slightly increased after 48 hours. This occurred due to the prolonged nucleation induction time of AM in WSC solutions caused by the inhibitory effect of WSC. Furthermore, the micelle formation of WSC can be related to the increased thermal equilibrium solubility of AM in WSC solution. A previous study reported that an increase in the equilibrium solubility of a drug by the presence of polymers decreases the apparent supersaturation level of the drug [29]. Therefore, AM concentration of 0.5 mg/mL was selected as a measure of the induction time to prevent the decrease in the apparent supersaturation level of AM.

### 3.2. Nucleation Induction Time Measurements

**Figure 5:**
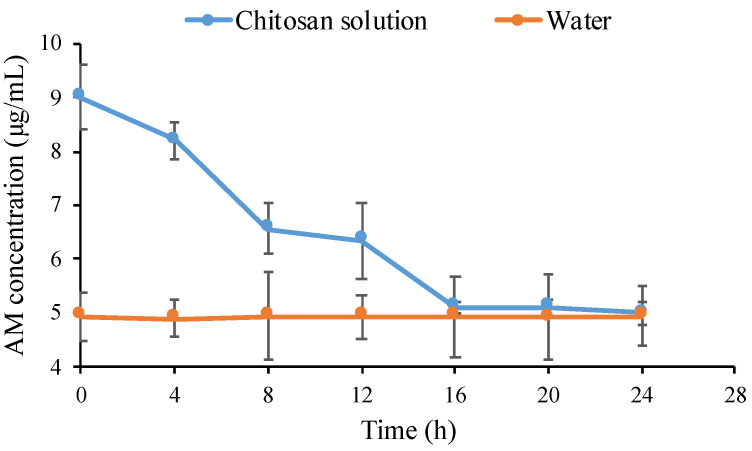
The concentration of AM in the water and WSC solutions (*n* = 3, mean ± standard deviation).

In the crystallization kinetics of the drug, the time required for the drug to form critical nuclei from the supersaturated solution is called nucleation time. Determination of drug crystal nuclei experimentally is not possible until a detectable size is achieved. Therefore, induction time is measured to describe the time required for the drug to form an observable change in the crystallization system from the starting point of drug supersaturation. It can be measured experimentally to describe the time required for the formation of an observable change in the crystallization system from the starting point of supersaturation. Figure 5 shows the induction time measurement of AM in WSC solution. The concentration of AM in the water rapidly decreased for one minute due to the crystal nuclei formation followed by crystal growth of AM. WSC maintained the initial supersaturation level of AM for 4 hours, indicating the ability of WSC to inhibit crystal nucleation. However, the AM concentration gradually decreased after 4 hours due to the recrystallization. The rate of crystal nucleation in the supersaturated solutions is dependent on the supersaturation level of the drug [30].

**Figure 6:**
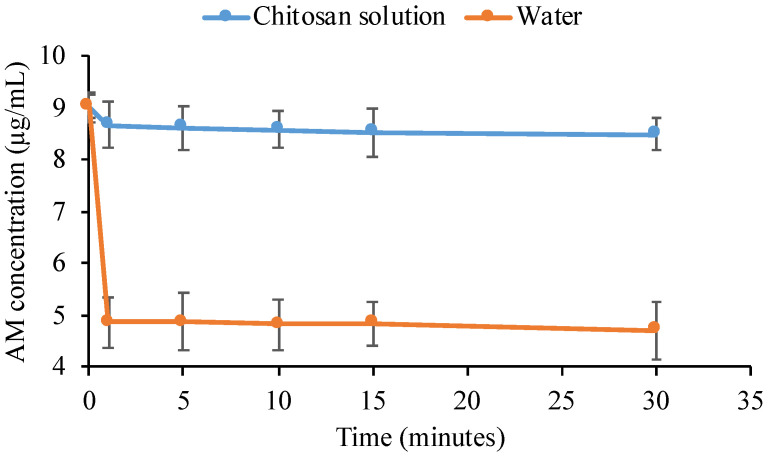
The concentration of AM in the water and WSC solutions for 30 min (*n* = 3, mean ± standard deviation).

The nucleation induction time was evaluated at the beginning of measurement at intervals of 1, 5, 10, 15, and 30 min, as shown in Figure 6, to determine the ability of chitosan to inhibit AM crystal nucleation. The result showed that chitosan maintained the initial supersaturation level of AM for 30 min, indicating the inhibitory effect of WSC on AM crystal. The objective of the present study was to clarify the impact of WSC on the growth and nucleation of AM from supersaturated solutions. Based on these data, it was observed that the WSC solution could effectively hinder the crystal growth and reduce nucleation of AM; thus, elevated levels of supersaturation could be maintained for extended periods of time. Polymeric additives have been widely utilized in supersaturated formulations because of their ability to inhibit drug crystallization [10]. It has been reported that polymers stabilize drugs in supersaturated solutions by intermolecular interactions between drugs and polymers, thus inhibiting drug crystal nucleation in the long term [12]. Therefore, WSC could be a potential polymer that maintained levels of AM supersaturation after dissolving in the water.

### 3.3. FT-IR Spectroscopy Analysis

**Figure 7:**
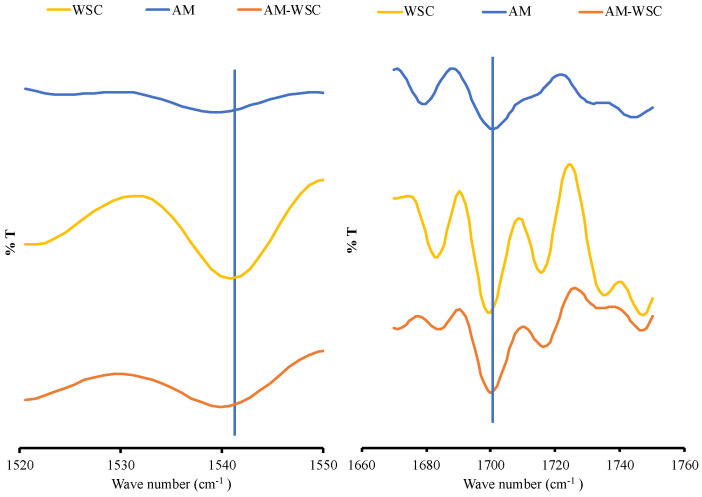
FT-IR spectrum of each sample in the range of 1520−1760 cm^−1^.

It is difficult to examine the infrared spectra of each sample in aqueous solutions because water can absorb infrared radiation. Therefore, to obtain the FT-IR spectra of each sample in an aqueous solution as shown in Figure 7, the FT-IR difference spectra were subtracted from the FT-IR spectrum of pure water. A previous study showed FT-IR spectra of chitosan at 1788 cm^−1^ and 1464 cm^−1^, which were indicated for amide I and amide II bonds, respectively. However, the amide group in the WSC has been totally lost due to the deacetylation reaction. Meanwhile, the peak at around 1700 cm^−1^ and 1500 cm^−1^ appeared, which represented the peak of -NH2 primer and amide deformation. The AM–WSC interaction showed a slight shift at around 1700 cm^−1^ and 1500 cm^−1^, which was attributed to -NH_2_ primer and amide deformation of WSC [24]. These shifts in the peaks suggest the possible intermolecular interaction between nitrogen from amine primer of WSC as an acceptor of hydrogen and the proton of AM as a donor hydrogen. However, the spectra are not so clear because the concentration of all samples, specifically AM, was very low. This makes it necessary to further investigate AM–WSC to clarify the effect of the interaction on the supersaturated solution of AM.

### 3.4. NMR Analysis

**Figure 8:**
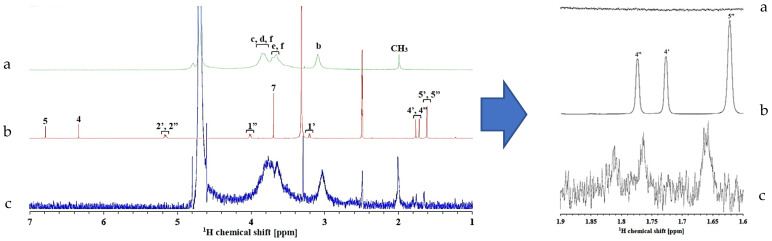
(**Right**) Expanded and (**left**) full ^1^H NMR spectra of (**a**) WSC, (**b**) AM, and (**c**) AM–WSC peak region in the WSC solution.

To confirm the molecular state of AM and the interaction of AM–WSC in the supersaturated solution, ^1^H NMR was also performed. The molecular-level characterization of supersaturated solutions is necessary to exhibit the mechanism of the inhibition of drug crystal nucleation because the formation of crystal nuclei in the drug-supersaturated solutions occurs on a tiny scale. NMR is a valuable technique for the characterization of the molecular states of drugs and additives in a solution. The chemical shifts and peak widths of the drug in the NMR spectra reflect their chemical environment and molecular mobility, which were used to evaluate the intermolecular interactions between drugs and polymers. The analysis of NMR was performed for the proton (^1^H-NMR), and the ^1^H NMR spectra of each sample are shown in Figure 8. The peak assignments of AM and WSC were assigned as described in the previous reports [31,32]. The AM peaks were shifted upfield by the addition of WSC in the supersaturated solutions of AM, specifically for H4 and H5 peaks. The AM peaks were significantly broadened, indicating mobility suppression of AM in the WSC solution. Thus, AM mobility was suppressed in the presence of WSC. Meanwhile, the WSC peaks were also shifted downfield from WSC at 3.103 ppm to AM–WSC at 3.045 ppm. These results indicated that the proton of WSC formed intermolecular interactions with the carbonyl group of AM, leading to crystallization inhibition of AM from supersaturated solution. The chemical shifts and differences in each sample are summarized in Table 1.

### 3.5. In Silico Study

**Figure 9:**
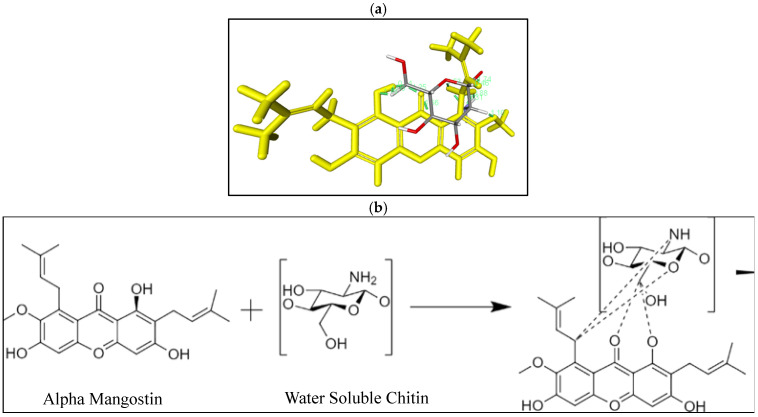
(**a**) Three-dimensional and (**b**) two-dimensional visualization of alpha-mangostin (AM) with water-soluble chitosan (WSC).

The interaction between AM and WSC was predicted using the ligand–ligand method to determine the hydrogen bonding, binding energy (Ei), and distance of each contact. The result of in silico study is shown in Figure 9 below.

The visualization screening showed the presence of unconventional hydrogen bonding in three interactions between AM and WSC. The carbonyl and hydroxyl functional groups from AM become the potential acceptor and donor of hydrogen bonds, respectively.

The non-conventional hydrogen bonding was observed on carbonyl groups from AM with hydrocarbon groups of WSC. Based on interaction distance, a wide range with typical values for WSC of 1.11 Amstrong was discovered. This showed that WSC has a low interaction distance that can produce strong interactions and stability.

According to the energy binding value, the AM interaction with WSC had energy binding (Ei) values of −3.3 Kcal/mol for WSC, showing that WSC can interact with AM. The low energy binding has a direct relationship with the lowest activation energy, which triggers spontaneous reactions. These results showed that the crystallization inhibition of AM in WSC solution can be attributed to the interaction between both in a supersaturated solution.

### 3.6. Viscosity Measurement

The results of the viscosity measurement are shown in Table 2.

The result showed that the addition of WSC into the water did not significantly change the viscosity of the solution, where all samples have a value of 4.22–5.34 cps. This indicated that crystallization inhibition of AM was not affected by increasing the viscosity due to the addition of polymer into water. Therefore, the crystallization inhibition of AM from supersaturated solution can be attributed to the interaction between AM and WSC.

## 4. Discussion

Drug supersaturation is a strategy to improve the absorption of the drug, specifically for those classified as Class II or IV by the Biopharmaceutics Classification System; however, crystallization is always observed in this method. Therefore, the addition of polymers such as water-soluble chitosan is needed to inhibit drug crystallization, maintaining the supersaturated state of the drug for a long time with preferable absorption.

**Figure 10:**
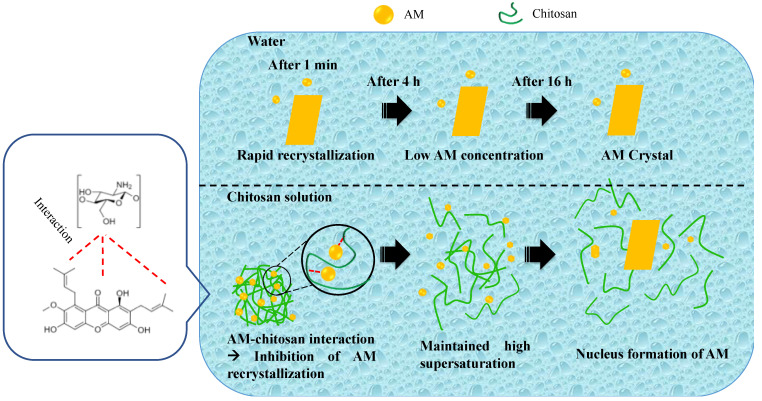
Schematic illustration of crystallization inhibition of AM from the supersaturated solution.

Nucleation time measurement has been proposed in recent years and used to predict the formation of drug crystal nucleation from the supersaturated solution. The previous study reported that crystal nucleation from supersaturated solutions is caused by the self-association of solute drugs and subsequent reorientation of their molecules. As shown in Figure 10, the AM concentration rapidly decreased after one minute, indicating it started to form self-associates in the AM-supersaturated solution due to its crystallization. Although the crystallization of AM occurred from the supersaturated solution, WSC effectively inhibited AM nucleation in the long term. Based on the nucleation theory, nucleation inhibition of drugs by polymers can be due to the elimination and/or inhibition of the reorientation of the self-associated drug. Therefore, it can be hypothesized that WSC prevented the formation of AM crystal nuclei by inhibiting the reorientation of AM self-associates.

The inhibitory effect of HPMC on the crystal nucleation of the drug has been reported in previous studies. The results showed that the crystal nucleation induction time of the drug was significantly delayed due to the intermolecular interaction between drug–HPMC, leading to the molecular mobility suppression and crystallization inhibition of the drug from the supersaturated solution [33]. This crystallization inhibition in amorphous solid dispersions has also been discussed, where the addition of polymer contributed to molecular mobility suppression of amorphous drugs, causing recrystallization inhibition [34,35]. In the NMR study, intermolecular interactions between the proton from WSC with the AM were observed. The hydrogen interaction between the carbonyl group of AM with the proton of WSC was also observed in the in silico study. It was discovered that WSC formed a hydrogen interaction with AM, which led to the molecular mobility suppression of AM to maintain high supersaturation. A previous investigation also stated that the molecular mobility suppression of drugs occurred due to an increase in the viscosity caused by polymer addition in the solution. In this study, the viscosity of the WSC solution was not significantly different compared to water (without WSC). This result showed that crystallization inhibition of AM from supersaturated solution can be attributed to the interaction between the carbonyl group of AM with the proton of WSC.

## 5. Conclusions

Induction time measurement revealed that a high concentration of AM was maintained in the WSC solution. Molecular-level characterization obtained by FT-IR, NMR, and in silico study also showed that WSC inhibited AM crystal nucleation in the supersaturated solutions by forming hydrogen bond interactions between the AM with WSC. This interaction led to the molecular mobility suppression of AM, which contributed to preventing AM crystallization and maintaining the supersaturated state of AM for a long time. This study provided fundamental insight into the selection of additives for the design of supersaturated formulations for optimized oral absorption of poorly water-soluble drugs.

## Figures and Tables

**Table 1:** Chemical shifts of H4 and H5 peaks of AM and Hb of WSC in the WSC solutions (500 μg/mL) and the difference in chemical shift compared to that in water.

Sample	Solution	Chemical Shift (ppm)			Different in the Chemical Shift (ppb)		
**Peak**		H4′	H4″	H5′, H5″	ΔH4′	ΔH4″	ΔH5′, H5″
AM	Water	1.774	1.727	1.622			
AM	WSC	1.810	1.764	1.660	36	37	38
**Peak**		Hb			ΔHb		
WSC	WSC	3.103					
WSC-AM	WSC	3.045			58		

**Table 2:** Result of the physical evaluation of the sample.

Sample	Viscosity (cps)
WSC	5.34
AM–WSC Water	5.34 4.22

## Data Availability

Not applicable.
